# The Min system and other nucleoid-independent regulators of Z ring positioning

**DOI:** 10.3389/fmicb.2015.00478

**Published:** 2015-05-13

**Authors:** Veronica W. Rowlett, William Margolin

**Affiliations:** Department of Microbiology and Molecular Genetics, University of Texas Medical School at Houston, Houston, TX, USA

**Keywords:** bacterial cell division, divisome, Min system, FtsZ, Z-ring positioning

## Abstract

Rod-shaped bacteria such as *E. coli* have mechanisms to position their cell division plane at the precise center of the cell, to ensure that the daughter cells are equal in size. The two main mechanisms are the Min system and nucleoid occlusion (NO), both of which work by inhibiting assembly of FtsZ, the tubulin-like scaffold that forms the cytokinetic Z ring. Whereas NO prevents Z rings from constricting over unsegregated nucleoids, the Min system is nucleoid-independent and even functions in cells lacking nucleoids and thus NO. The Min proteins of *E. coli* and *B. subtilis* form bipolar gradients that inhibit Z ring formation most at the cell poles and least at the nascent division plane. This article will outline the molecular mechanisms behind Min function in *E. coli* and *B. subtilis*, and discuss distinct Z ring positioning systems in other bacterial species.

## Introduction

In bacteria, several proteins assemble at the cell center (midcell) to form the divisome, or cell division machine (Martos et al., 2012). At the start of divisome formation, FtsZ, a homolog of eukaryotic tubulin, polymerizes and forms a ring-like structure, the Z ring, at midcell (Bi and Lutkenhaus, 1991). The Z ring functions as a scaffold for cell division proteins and is an obvious target for regulating the site of cytokinesis. Decades of research in *E. coli* and *B. subtilis* have led to insights into the molecular mechanisms of NO and the Min system, which negatively regulate Z ring formation by preventing Z rings from forming over nucleoids or at cell poles, respectively. However, it is likely that other regulators contribute to Z ring positioning in these bacteria (Rodrigues and Harry, 2012). Recently, unique negative and positive regulators of Z ring formation have been identified in bacteria that lack NO and/or Min systems, highlighting the diversity of division site selection mechanisms.

## Minicells and the Min System

Several decades ago, Adler et al. (1967) identified small, nucleoid free *E. coli* cells described as minicells. These minicells do not divide, but remain metabolically active for hours. Minicells also form in *B. subtilis* and other bacteria (Reeve et al., 1973). Historically, purified minicells were used to produce radiochemically pure proteins from high-copy plasmids that partition into minicells and could be selectively labeled in purified minicell preparations (Roozen et al., 1971). More recently, minicells have been useful for viewing cell surface structures by cryo-electron tomography (Liu et al., 2012; Hu et al., 2013) and are also being developed as safe cellular systems for antigen delivery to tumor cells (MacDiarmid and Brahmbhatt, 2011; Carleton et al., 2013).

**FIGURE 1:**
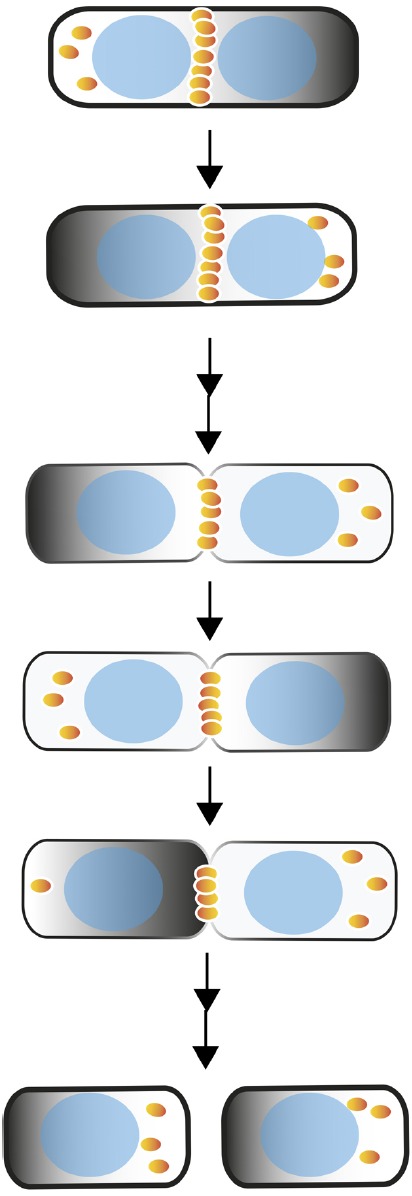
**Negative spatial regulation of Z ring positioning in *E. coli* by the Min system.** Shown are Min oscillations (dark gradients), dynamics of FtsZ (orange spheres) and eventual assembly as a Z ring at the midpoint of a wild-type cell through the process of cell division and partitioning of the Min system to daughter cells. Nucleoids are depicted as blue ovals; passage of time in seconds is represented by an arrow, passage of time in minutes by a double arrow.

In *E. coli*, Min proteins localize to the cell poles and function to prevent Z rings from forming near those poles (Figure 1; Bramkamp and van Baarle, 2009). In *B. subtilis*, the Min system has a somewhat different role (see next section). When Min proteins are deleted or non-functional, Z rings are able to form both at correct midcell locations and at cell poles, resulting in minicells and cells of heterogeneous length, as not all Z rings are functional to complete division (de Boer et al., 1989; Yu and Margolin, 1999). One of the Min proteins, MinC, directly interacts with FtsZ and inhibits FtsZ polymerization (Hu et al., 1999; Dajkovic et al., 2008; Shen and Lutkenhaus, 2009, 2010). As described below, MinC also interacts with partner proteins that localize it to cell poles, creating a gradient of MinC that is highest at cell poles and lowest at midcell (Monahan and Harry, 2012).

## Min Systems of *B. subtilis* and *E. coli*

In *B. subtilis*, DivIVA localizes the Min proteins to the poles of the cell (Cha and Stewart, 1997; Edwards and Errington, 1997). DivIVA functions as a generic cell-pole targeting protein, as it senses negative membrane curvature in a wide variety of species, including fission yeast (Edwards et al., 2000; Lenarcic et al., 2009). As a result, DivIVA relocalizes from cell poles to the new septum because of its sharp curvature. MinJ acts as an adaptor protein between polarly-targeted DivIVA and MinD, efficiently recruiting MinD to cell poles, where it binds the cytoplasmic membrane via its C-terminal amphipathic helix (Szeto et al., 2002). MinD binds MinC directly, and by doing so recruits the Z ring inhibitor MinC to the cell poles (Bramkamp et al., 2008; Patrick and Kearns, 2008). The Min system of *B. subtilis* was originally believed to migrate to existing cell poles to form a static bipolar gradient, with polar MinC inhibiting assembly of Z rings only at cell poles. However, more recent evidence indicates that Min proteins are recruited to midcell prior to septation and formation of new cell poles (Gregory et al., 2008). It is thought that localization of Min proteins to midcell prevents more than one Z ring from forming there, and plays a role in establishing a new bipolar gradient in daughter cells (Gregory et al., 2008; van Baarle and Bramkamp, 2010). The Min system thus does not seem to identify midcell as much as promote efficient use of the midcell site (Rodrigues and Harry, 2012).

**FIGURE 2:**
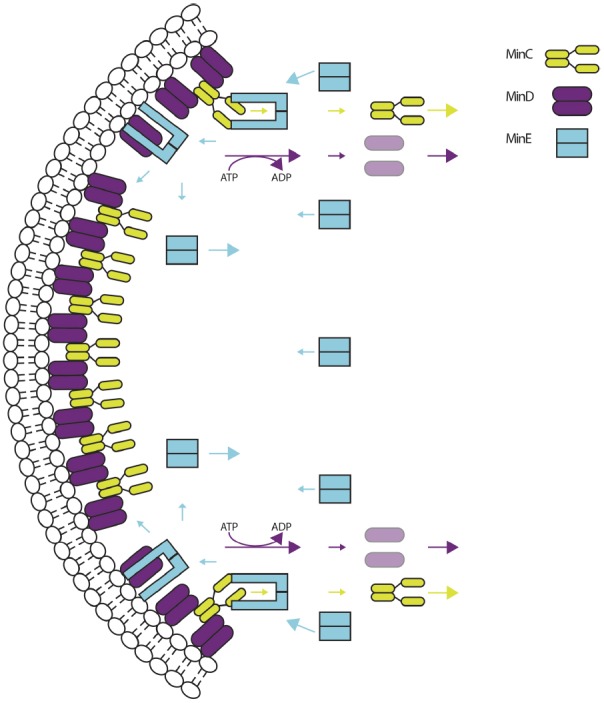
**The molecular mechanism of Min oscillation.** Membrane-bound complexes of MinC (chartreuse) and MinD (purple) are targeted by MinE (cyan). MinE dimers change conformation and bind MinD and the membrane, displacing MinC and stimulating the ATPase activity of MinD and its removal from the membrane. MinC and MinD-ADP move toward the opposite pole and begin another cycle of oscillation. MinE can stay membrane bound to remove more MinCD complexes, or change conformation and follow MinD to the opposite pole. Adapted from Rowlett and Margolin (2013).

The Min system in *E. coli* differs from that of *B. subtilis* because *E. coli* lacks DivIVA and instead contains a third Min protein, MinE. Because they lack DivIVA, *E. coli* cells need to establish a MinC bipolar gradient without the benefit of a polar targeting protein. As in *B. subtilis*, *E. coli* MinD directly binds the membrane and directly binds MinC (de Boer et al., 1991; Hu and Lutkenhaus, 2003). The MinE protein is critical for targeting the MinCD complex to the cell poles (see below). MinE forms a ring and causes MinD to be removed from one pole and migrate to the opposite pole (Raskin and de Boer, 1997; Hu et al., 2002). The dynamics between MinD and MinE create an oscillating system, in which MinC is a passenger (Figure 2; Hu and Lutkenhaus, 1999; Raskin and de Boer, 1999a,b). As a result of the oscillation, where most of the time is spent at the cell poles with only a short transit (typically 10 s) between them, the average position of MinC over time is at the poles and not at midcell, which leaves midcell as the least inhibited location for FtsZ assembly (Figure 1).

Although it is fairly well understood how the Min systems of *E. coli* and *B. subtilis* position Z rings during vegetative growth, relatively little is known about the requirements for placement of Z rings near the cell poles in preparation for asymmetric septation in *B. subtilis* and other Gram positive endospore-forming bacilli such as clostridia. A clue comes from the reconstitution of *E. coli*’s oscillating Min system in *B. subtilis*. Fluorescently-tagged MinD and MinE from *E. coli* oscillate from cell pole to cell pole when produced in *B. subtilis* cells deleted for native *minD* (Jamroškovič et al., 2012), consistent with the self-contained nature of the MinDE oscillator (Ramirez-Arcos et al., 2002). Interestingly, this artificial oscillation inhibited sporulation, likely by preventing the assembly of Z rings near cell poles that is a hallmark of endospore formation in *B. subtilis*. This suggests that a constitutive oscillatory Min regime, while optimal for medial cell division in cells like *E. coli* that only grow vegetatively, may be problematic in cells that need to switch to asymmetric division for endospore formation (Barák, 2013). The presence of both MinDE and MinDJ/DivIVA in spore-forming clostridia may point to an ancestral Min system that exploited the advantages of each system, perhaps for switching from a vegetative oscillatory mode to a sporulation-induced static mode.

## Molecular Mechanism of Min Oscillation in *E. coli* Cells

The MinC protein has two distinct domains that synergistically inhibit polymerization of FtsZ. The N-terminal half of the protein is sufficient to antagonize the longitudinal interactions of FtsZ subunits within a protofilament, whereas the C-terminal half inhibits the lateral interactions between FtsZ protofilaments (Hu and Lutkenhaus, 2000; Shiomi and Margolin, 2007; Shen and Lutkenhaus, 2009, 2010). The C-terminus of MinC binds to MinD, which is required for the full inhibition of FtsZ lateral interactions, and is important for MinC dimerization (Hu and Lutkenhaus, 2000, 2003). MinD is a ParA family ATPase that contains a C-terminal membrane targeting sequence (MTS) as mentioned above (de Boer et al., 1991; Hu and Lutkenhaus, 2003). When bound to ATP, MinD dimerizes and associates with the membrane (Szeto et al., 2002; Hu and Lutkenhaus, 2003). MinC and MinD form copolymers of alternating MinC and MinD dimers that are similar to eukaryotic septins (Ghosal et al., 2014; Conti et al., 2015). MinCD complexes are removed from the membrane by MinE (Raskin and de Boer, 1997).

In *E. coli* cells lacking MinE, MinCD binds to the membrane and inhibits Z ring formation throughout the cell, resulting in lethal filamentation (de Boer et al., 1989). Thus, MinE is essential for the MinCD complex to assume its role as a spatial regulator. MinE packs many activities into its compact 88 amino acid size. First, it harbors an amphipathic helix at its N-terminus that serves as an MTS, and direct membrane interaction is important for its function (Ma et al., 2003; Hsieh et al., 2010). Earlier mathematical models did not account for this membrane binding, but more recent models have (Schweizer et al., 2012; Bonny et al., 2013). Upon recognizing MinD, dimers of MinE change conformation, bind MinD, displace MinC, and stimulate the ATPase activity of MinD, causing monomerization of MinD and its removal from the membrane (Figure 2; Raskin and de Boer, 1997; Hu et al., 2002; Suefuji et al., 2002; Lackner et al., 2003; Loose et al., 2011; Park et al., 2011). Once dislodged from the membrane, MinD-ATP is regenerated from MinD-ADP by ADP-ATP exchange and immediately re-binds the membrane (Huang et al., 2003). However, it will tend to bind the membrane far from its most recent complex, because the MinE complex that recently removed it can remain on the membrane via its MTS (Loose et al., 2011; Park et al., 2011). This allows MinE to remove other bound complexes of MinCD at the original site, including those that recently re-bound, before changing back to its original conformation and migrating to the opposite pole (Park et al., 2011).

In support of this molecular mechanism, MinE always lags behind MinD. Because MinD-ATP binds to the membrane cooperatively, newly formed MinD-ATP will form a large complex as far away as possible from the original site, which happens to be the opposite pole of a normal sized *E. coli* cell. MinE, with no substrate remaining at the original site, diffuses through the cytoplasm, and possibly the membrane, until it binds to the edge of the newly assembled MinD complex at the opposite pole. *In vivo*, this is observed as a ring of MinE at the edge of the polar zone of MinD. Because the MinD polar zones are large and extend far from the cell pole, MinE rings are often observed near midcell, where they then follow the edge of the MinD polar zone as it shrinks toward the pole (Raskin and de Boer, 1997).

While Min proteins oscillate between cell poles, proteins that are recruited in the early stages of cell division, including FtsZ, ZipA, ZapA, and ZapB, also oscillate oppositely from the Min system with the same period as the Min system (Thanedar and Margolin, 2004; Bisicchia et al., 2013; Tonthat et al., 2013). ZipA and FtsA function to tether the Z ring to the inner membrane (Pichoff and Lutkenhaus, 2005), and ZipA has also been shown to bundle FtsZ protofilaments *in vitro* (Hale and de Boer, 1999; Hale et al., 2000). ZapA recruits ZapB to the Z ring and these proteins function to stimulate and stabilize Z ring formation (Gueiros-Filho and Losick, 2002; Galli and Gerdes, 2010; Buss et al., 2013). As ZipA and ZapA bind to FtsZ directly, and ZapB binds to ZapA, all three proteins are dependent on the Z ring for their localization. Therefore, their counter-oscillatory behavior is likely caused by the periodic assembly and disassembly of Z ring precursor complexes in response to oscillating waves of MinC (Thanedar and Margolin, 2004; Bisicchia et al., 2013).

## Other Factors Influencing Min Oscillation

The oscillation of Min proteins can vary in response to changes in growth conditions, protein levels, and the cell cycle. For example, increased temperatures will shorten the period of Min oscillation from about a minute to several seconds (Touhami et al., 2006). Higher levels of MinD relative to MinE reduce MinD ATPase activity and lengthen the oscillation period, whereas lower MinD:MinE ratios increase MinD ATPase activity and shorten the period (Raskin and de Boer, 1999b; Hu et al., 2002), which is consistent with MinE as the driver of MinD dynamics. During later stages of cell division when the division septum is closing, MinD begins to “pause” at midcell prior to the formation of two separate oscillating systems, one in each daughter cell (Juarez and Margolin, 2010). Intriguingly, MinD is often observed to localize at opposite sides of the developing septum prior to doubling. This phenomenon is likely a result of the doubling in pole-to-pole distance prior to division. The pausing of MinD at midcell is likely required for equal partitioning of MinD into daughter cells and has been simulated mathematically (Di Ventura and Sourjik, 2011). This behavior might also prevent new Z rings from forming adjacent to existing rings, similar to the septal localization observed for Min proteins in *B. subtilis* as described above (Gregory et al., 2008; van Baarle and Bramkamp, 2010). This idea is supported by evidence that MinC also exhibits similar septal pausing (Hu and Lutkenhaus, 1999).

In addition to the pausing phenomenon, Min oscillation is strongly influenced by cell geometry. *E. coli* cells of normal length have a pole-to-pole pattern of Min localization, which is constrained by the essentially one-dimensional cellular rod shape (Raskin and de Boer, 1999b). If cells become elongated, such as in division-defective mutants, oscillation no longer extends from one pole to the other because the pole-to-pole distance becomes too long. Instead, the pattern changes to multiple (2 or more) oscillating units, some of which form traveling waves, in which MinD alternates from traveling through the membrane to traveling through the cytoplasm between poles (Raskin and de Boer, 1999b; Hu and Lutkenhaus, 2001; Bonny et al., 2013). The result is that zones of MinCD appear and disappear throughout the filamentous cell with ∼7 μm spacing (Raskin and de Boer, 1999b; Meinhardt and de Boer, 2001). It is likely that these MinCD zones help restrict where Z rings form distal to cell poles within these filaments.

Min proteins also respond to changes in cell shape, indicating that oscillation is not restricted to a symmetrical pattern. For example, in *E. coli* cells such as *rodA* mutants that grow and divide as spheres, MinD and MinE will migrate from one location to another on the cell periphery in a seemingly disorganized pattern, although there is a strong bias for migration down a long axis (Corbin et al., 2002). This behavior has been simulated by mathematical models (Huang and Wingreen, 2004). Such round cell mutants divide in alternating perpendicular planes, much like Staphylococci except only in two dimensions instead of three; if the Min system is inactivated in these mutant cells, their division becomes highly irregular (Begg and Donachie, 1998; Corbin et al., 2002). When round cells divide, they pinch and form a long axis, thus establishing Min oscillations parallel to the growing septum. This pattern will then define the midpoint of future perpendicular Z ring or arc (Pas et al., 2001), which explains how this alternating division pattern can occur (Corbin et al., 2002). Other evidence for geometric control of Min oscillation comes from branched *E. coli* cells, where Min proteins travel in a clockwise or counterclockwise direction between cell branches (Varma et al., 2008).

Membrane curvature and/or phospholipid composition likely serve as physical cues for localization of DivIVA and MinD (Barák and Muchová, 2013). The anionic phospholipid cardiolipin contributes to membrane curvature, and is enriched both at cell poles and the septum of dividing *E. coli* and *B. subtilis* cells (Mileykovskaya and Dowhan, 2000; Kawai et al., 2004). Phosphatidylglycerol is also enriched at *E. coli* cell poles and may contribute to the anionic nature of polar membranes, which seems to be important for binding of MinD and MinE (Mileykovskaya et al., 2003; Mazor et al., 2008; Renner and Weibel, 2012; Oliver et al., 2014; Vecchiarelli et al., 2014). In mutants lacking phosphatidylethanolamine (PE), MinD does not oscillate in organized zones but instead assembles and disassembles dynamically at peripheral foci throughout the cell, probably because the levels of anionic phospholipids are too high (Mileykovskaya et al., 2003). Cells lacking PE also divide poorly and exhibit spiral FtsZ patterns (Mileykovskaya et al., 1998), but is not yet known if these effects result from perturbations of the Min system or of other proteins that affect FtsZ assembly and function. In *B. subtilis*, on the other hand, specific phospholipids do not seem to be involved in the polar targeting by DivIVA (Lenarcic et al., 2009).

## Cell-free Reconstitution of Min Protein Dynamics

**FIGURE 3:**
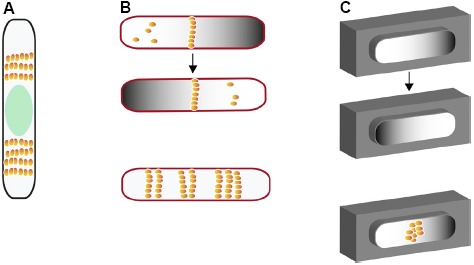
**Different model systems for investigating nucleoid-independent Z ring positioning in *E. coli*. (A)** Multiple Z rings in cells lacking both Min and topoisomerase IV with large nucleoid-free regions on either side of unpartitioned nucleoid (green oval). **(B)** (top) Min oscillations and FtsZ positioning in nucleoid-free maxicells (outlined in red); (bottom) Z ring positioning in maxicells containing FtsZ that is unresponsive to MinC. **(C)** Cell-free oscillation of MinCDE in artificial cell-like compartments coated with a lipid bilayer before (top and middle) and after addition of purified FtsZ (bottom). FtsZ is depicted as orange spheres.

The Min system has been an attractive subject for generating mathematical models to better understand Min protein dynamics (Kruse et al., 2007; Bonny et al., 2013). Such models of the Min system have both confirmed *in vivo* data, and have been used to predict patterns later confirmed using *in vivo* experiments (Bonny et al., 2013). These models implied that MinD and MinE were sufficient for the oscillatory behavior, and as proof of this, purified MinD and MinE were shown to self-organize into wave patterns on supported lipid bilayers (SLBs) in the presence of ATP (Loose et al., 2008). MinD first binds ATP and forms a homogenous layer on the bilayer, and when MinE is added, planar waves form, with MinD at the leading edge and MinE at the trailing edge (Loose et al., 2008). This nicely mimics the lagging behavior of MinE observed in *E. coli* cells (Hale et al., 2001). Reconstitution of FtsZ and MinC on SLBs uncovered the molecular mechanism of MinC inhibition of FtsZ by demonstrating that MinC can both bind FtsZ to prevent monomers from incorporating into polymers, and remove monomers from existing polymers (Arumugam et al., 2014). When FtsZ was reconstituted on the SLBs with MinDE, with and without MinC, MinC was shown to be required for spatial organization of FtsZ on MinDE waves (Arumugam et al., 2014). Most convincingly, MinCDE oscillation from pole to pole was reconstituted in rod-shaped compartments covered with membranes, as was FtsZ assembly at the midpoint of those compartments (Figure 3C; Zieske and Schwille, 2013, 2014).

## The Min System Acts Independently of the Nucleoid but May Influence Chromosome Partitioning

Certain chromosome partitioning mutants of *E. coli*, such as those inactivating Topoisomerase IV or the MukBEF condensing complex, result in a relatively high percentage of anucleate cells (Hiraga et al., 1991; Stewart and D’Ari, 1992; Hayama and Marians, 2010). Importantly, Z rings are positioned near the center of anucleate cells, indicating that chromosomes, though active for NO, are not essential for Z ring positioning (Sun et al., 1998). Indeed, mathematical models for Min-mediated centering of the Z ring assume that Min proteins are sufficient without any other macromolecules needed. Moreover, Min oscillations occur independently of nucleoid structural changes (Sun and Margolin, 2001), and Z rings can be centered by the Min system alone in the reconstitution experiments described above. Finally, *E. coli* maxicells, which are generated by UV-mediated destruction of the chromosome and are thus nucleoid-free, retain an oscillating Min system and also support Z ring formation at midcell. This supports the *in vitro* reconstitution data and suggests that the Min system alone can restrict Z rings to midcell in *E. coli* (Figure 3B; Pazos et al., 2014).

One question in the field is what advantages might the ATP-burning Min oscillation confer upon *E. coli*, considering that the *B. subtilis* Min system does not oscillate. Several studies have suggested that *E. coli* cells lacking Min systems have defects in chromosome partitioning (Bernander et al., 1989; Åkerlund et al., 1992, 2002). As the driving forces for chromosome partitioning in *E. coli* are unknown, this brings up the possibility that the Min oscillation somehow aids this process. Recent evidence suggests that MinD can bind chromosomal DNA directly, and therefore tether the chromosome to the membrane (Di Ventura et al., 2013), which might influence chromosome partitioning (Schofield et al., 2010).

Even when it is not oscillating, the *B. subtilis* Min system is also linked to chromosome replication. Another ParA family ATPase called Soj binds to chromosomal DNA and the DnaA initiator protein to activate DNA replication. However, Soj is also present as an inactive form that binds to MinD at cell poles, which prevents Soj activation of DNA replication (Murray and Errington, 2008).

## Additional Regulators of Z ring Positioning in *E. coli* and *B. subtilis*

Recent evidence suggests that spatial regulators of Z ring positioning other than NO and Min exist in *E. coli* and *B. subtilis*. In *E. coli* cells, deletions of the Min system and SlmA, the mediator of NO, are synthetically lethal when cells are grown in rich medium (Wu and Errington, 2004; Bernhardt and de Boer, 2005). Removing both systems results in too many potential locations for Z rings to form Figure 3A, preventing assembly of a single coherent ring at midcell (Yu and Margolin, 1999; Bernhardt and de Boer, 2004). However, in minimal medium, cells that lack Min proteins and NO can survive and divide quite well, although the reason for the growth medium-dependence is not clear (Bernhardt and de Boer, 2005). Upon further investigation, cells lacking both NO and Min had more precise Z ring placement and produced fewer minicells than cells that only lacked Min (Bailey et al., 2014). These findings suggest that when both systems are not present, other factors contribute to Z ring positioning (Cambridge et al., 2014).

These factors may involve the nucleoid itself. For example, a positive regulatory system that involves the Ter macrodomain region of the chromosome has been implicated in Z ring positioning, as it occupies the center of the nucleoid during Z ring positioning (Bailey et al., 2014). In this system, MatP serves to connect the Ter macrodomain to the divisome through interaction with ZapB (Espéli et al., 2012), and this network is involved in regulating septal constriction (Buss et al., 2015). Cells that lack all known systems still exhibit a bias for Z rings localizing to midcell, although with lower precision and accuracy, indicating that other overlapping factors help to position Z rings (Bailey et al., 2014). *B. subtilis* cells that lack NO and Min proteins also precisely position Z rings, but Z ring formation is delayed, indicating their importance for Z ring formation at the proper time of the cell cycle (Rodrigues and Harry, 2012). Positive markers for Z ring formation in *B. subtilis* cells are also hypothesized (Moriya et al., 2010). In their “midcell potentiation” model, Moriya et al. (2010) proposed that as chromosome replication progresses, it acts along with NO and other factors to license midcell for Z-ring assembly. Essential chromosomal DNA replication proteins such as DnaX of *E. coli* (Bates and Kleckner, 2005) and DnaB of *B. subtilis* (Imai et al., 2000) localize at or near midcell. What first attracts these proteins to midcell is a key unanswered question.

## Negative Spatial Regulation of Z ring Formation in *Caulobacter*

Like many other bacterial species, *Caulobacter crescentus* lacks both NO and Min systems, but contains a protein that restricts Z ring formation to midcell (Thanbichler and Shapiro, 2006). MipZ is a member of the ParA-like family of ATPases that includes MinD, and is conserved in α-proteobacteria (Thanbichler and Shapiro, 2006). In *C. crescentus,* MipZ binds to the chromosomal replication origin (*oriC*) and forms a complex with proteins that are involved in chromosome partitioning. Prior to replication and partitioning, *oriC* and bound MipZ are at one cell pole, and FtsZ is at the opposite pole, farthest from MipZ. Upon chromosome replication, the duplicated *oriC* migrates to the opposite cell pole, forming a bipolar gradient (Thanbichler and Shapiro, 2006). Unlike MinD, MipZ is able to directly interact with FtsZ and inhibit polymer formation, therefore the bipolar gradient of MipZ permits assembly of FtsZ at midcell, at the lowest MipZ concentration (Thanbichler and Shapiro, 2006; Kiekebusch et al., 2012). In contrast to *E. coli*, this seems to be a mechanism to position the Z ring in response to both spatial and cell cycle cues without the need for a separate NO system.

## Positive Spatial Regulators of Z ring Formation

**FIGURE 4:**
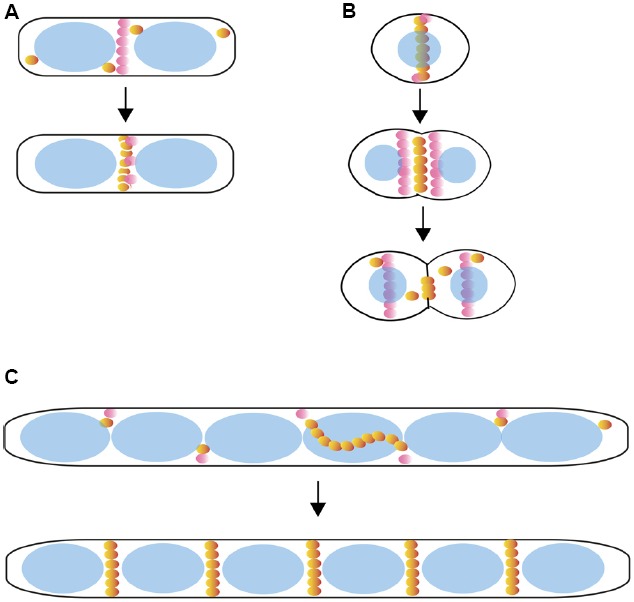
**Positive control of Z ring positioning.** Shown are models for positive spatial regulation of Z rings by PomZ in *Myxococcus xanthus* **(A)**; MapZ/LocZ in *Streptococcus pneumoniae* **(B)**; SsgB in *Streptomyces* aerial mycelia that are developing into spores **(C)**. A sample cell cycle progression is shown for each. The above regulators are represented by pink spheres, FtsZ as orange spheres, and nucleoids as blue ovals.

Until a few years ago, the known spatial regulators of the Z ring all acted to prevent Z ring formation at undesirable sites such as the cell poles or over the nucleoid. This negative regulation was satisfying because it generally exploited cell poles, which are a defined part of the cell that were once division sites, and did not need to invoke a midcell marker. Recently, however, several proteins that positively regulate Z ring formation have been identified in bacterial species that lack NO and/or Min systems. These proteins locate to the midcell division site and promote Z ring formation there, the very opposite of the Min/NO mechanism. The first reported example of a positive regulation system is the SsgA-SsgB pair in *Streptomyces coelicolor* (Willemse et al., 2011). SsgA-like proteins are present only in actinomycetes and are involved in forming multiple sporulation septa (Traag and van Wezel, 2008). *S. coelicolor* is able to grow vegetatively as mycelia in the absence of cell division, which enabled this study to be done in the absence of FtsZ. Notably, SsgA and SsgB localize to division sites between nucleoids in an *ftsZ* null mutant, indicating that their localization is independent of FtsZ (McCormick et al., 1994; Willemse et al., 2011). SsgA recruits SsgB to midcell, where SsgB likely promotes FtsZ polymerization into discrete Z rings and serves as a membrane tether for the rings (Willemse et al., 2011). During formation of aerial spores, multiple Z rings are recruited to SsgB foci, ultimately forming sporulation septa (Figure 4C).

In another example of what seems to be positive regulation, PomZ, a ParA family ATPase, positions Z rings in the *∂*-proteobacterial species known for its ability to swarm and produce fruiting bodies, *Myxococcus xanthus* (Treuner-Lange et al., 2013). *M. xanthus* cells lacking PomZ have classic defects in cell division that are hallmarks of aberrant Z ring positioning, including filamentation and the formation of minicells (Treuner-Lange et al., 2013). PomZ localizes to midcell prior to, and in the absence of, FtsZ Figure 4A). However, a direct interaction between PomZ and FtsZ has not yet been observed, indicating that other unidentified proteins are involved in bridging this connection and/or positioning the Z ring (Treuner-Lange et al., 2013).

Rod-shaped Actinobacteria have less precise Z ring placement when compared to *E. coli* and *B. subtilis* (reviewed in Donovan and Bramkamp, 2014). Although lacking Min and NO homologs, *Corynebacterium glutamicum* produces a homolog of ParA, PldP, which may play a role in localizing the Z ring (Donovan et al., 2010). PldP localizes to midcell early in the cell cycle, and inactivation of PldP results in an increased variation in cell length as well as minicell formation (Donovan et al., 2010). Further work is required to confirm the role of PldP in Z ring positioning.

Until recently, the only known spatial regulators of FtsZ were in naturally rod-shaped species, and it was unknown how new Z rings formed in cocci. However, a transmembrane protein in *Streptococcus pneumoniae*, called either LocZ (Localizing at midcell of FtsZ) or MapZ (Mid-cell-anchored protein Z) has been found to localize to midcell and contribute to Z ring positioning, cell shape, and division (Fleurie et al., 2014; Holečková et al., 2014). Cells lacking LocZ/MapZ are viable, but have cell shape defects and form minicells (Holečková et al., 2014). MapZ/LocZ localizes as rings at new division sites prior to the arrival of FtsZ (Figure 4B), and it was shown that MapZ interacts directly with FtsZ (Fleurie et al., 2014; Holečková et al., 2014). Once the midcell Z ring forms, MapZ/LocZ gradually move from midcell to the cell quarters, the sites of the next division (Fleurie et al., 2014; Holečková et al., 2014). Together, the evidence suggests that this regulator may stimulate FtsZ assembly at midcell. Homologs of LocZ/MapZ are present in streptococci, lactococci, and enterococci, suggesting that these species regulate Z ring positioning by a similar mechanism (Fleurie et al., 2014; Holečková et al., 2014). There has been no published report of FtsZ assembly *in vitro* from streptococci, perhaps because this FtsZ assembles poorly without a stimulatory factor.

## Conclusion and Perspectives

The wide varieties of Z ring positioning systems identified in recent years highlight the complexity and diversity of bacteria (Monahan et al., 2014). The two systems that are best understood are the negative regulators of Z ring placement, NO and Min. Further studies will be required to elucidate the mechanism of recently identified systems, and to identify factors that contribute to Z ring positioning that are currently unknown. A common theme among many known Z ring positioning systems is the involvement of ParA-like proteins, which are also involved in chromosome segregation (Lutkenhaus et al., 2012). Another important question for further study is how positive regulators of Z ring positioning are recruited to midcell, particularly as the midpoint of a rod shaped cell does not feature any known molecular marker. In the cases of PomZ and MapZ/LocZ, it remains possible that their positioning at midcell is indirectly orchestrated by negative regulatory factors that depend on cell poles as cues. On the other hand, there is evidence that round cells such as *Staphylococcus aureus* may directly use spatial cues derived from previous septation events. Prior division septa result in orthogonal belts of distinct cell wall material that encircle the cell, acting to mark those sites for several generations. It is thought that the intersection of these belts mark the site for new cell division events (Turner et al., 2010). NO also has a role in Z ring placement in *S. aureus*, as loss of NO perturbs Z ring placement (Veiga et al., 2011). It remains to be determined how widespread this type of mechanism is and which proteins help mediate assembly of Z rings in these systems.

### Conflict of Interest Statement

The authors declare that the research was conducted in the absence of any commercial or financial relationships that could be construed as a potential conflict of interest.
